# Prevalence and associations of symptoms of upper extremities, repetitive strain injuries (RSI) and 'RSI-like condition'. A cross sectional study of bank workers in Northeast Brazil

**DOI:** 10.1186/1471-2458-5-107

**Published:** 2005-10-11

**Authors:** Eliana M Lacerda, Luis C Nácul, Lia G da S Augusto, Maria Teresa A Olinto, Dyhanne C Rocha, Danielle C Wanderley

**Affiliations:** 1 London School of Hygiene and Tropical Medicine, Keppel Street, London WC1E 7HT, UK.; 2 Centro de Pesquisa Aggeu Magalhães – CPqAM/Fiocruz, Av. Moraes Rego s/n, Recife 50.670-420, Brazil.; 3 Programa de Pós-Graduação em Saúde Coletiva, Universidade do Vale do Rio dos Sinos, Av Unisinos 950, São Leopoldo 93022-000, Brazil.; 4 Faculdade de Ciências Médicas de Pernambuco, Universidade de Pernambuco, Rua Arnóbio Marques 310, Recife 50100-130, Brazil.

## Abstract

**Background:**

The repetitive strain injury syndrome (RSI) is a worldwide occupational health problem affecting all types of economic activities. We investigated the prevalence and some risk factors for RSI and related conditions, namely 'symptoms of upper limbs' and 'RSI-like condition'.

**Methods:**

We conducted a cross-sectional study with 395 bank workers in Recife, Northeast Brazil. Symptoms of upper limbs and 'RSI-like condition' were assessed by a simple questionnaire, which was used to screen probable cases of RSI. The diagnosis of RSI was confirmed by clinical examination. The associations of potential risk factors and the outcomes were assessed by multiple logistic regression analysis.

**Results:**

We found prevalence rates of 56% for symptoms of the upper limbs and 30% for 'RSI-like condition'. The estimated prevalence of clinically confirmed cases of RSI was 22%. Female sex and occupation (as cashier or clerk) increased the risk of all conditions, but the associations were stronger for cases of RSI than for less specific diagnoses of 'RSI-like condition' and symptoms of upper limbs. Age was inversely related to the risk of symptoms of upper limbs but not to 'RSI-like' or RSI.

**Conclusion:**

The variation in the magnitude of risk according to the outcome assessed suggests that previous studies using different definitions may not be immediately comparable. We propose the use of a simple instrument to screen cases of RSI in population based studies, which still needs to be validated in other populations. The high prevalence of RSI and related conditions in this population suggests the need for urgent interventions to tackle the problem, which could be directed to individuals at higher risk and to changes in the work organization and environment of the general population.

## Background

Repetitive strain injury (RSI) is an occupational disease that considerably impacts workers' lives and has significant socio-economic repercussions. The burden of RSI to people affected and the society are undeniably large. One third of workers' compensation costs in the US private industry are due to RSI[1], and the direct costs with compensation exceeds US$ 20 billion in the Washington State alone[2]. In the Netherlands, 8% of the whole working population take time off work because of RSI symptoms [3]. The Health & Safety Executive, a British institution responsible for the regulation of occupational risks to health, estimated self-reported work related musculoskeletal disorders to affect 448,000 people in 2003/04, corresponding to 1% of the population who has ever worked in Great Britain [4]. In the years 2001/02, it was estimated that 4.1 million full working days were lost due to work related musculoskeletal disorders in the United Kingdom. The estimated costs for employers associated with the condition was between £208 million and £221 million per year (1995/96 prices) [4].

Clinical, epidemiological and social aspects of RSI remain largely controversial in the medical literature [5-9]. Nevertheless, RSI has been widely shown to affect a considerable proportion of the adult population and workers in all levels of economic activities. Certain occupational groups have an increased risk of developing RSI. Among these are bank workers, particularly cashiers, whose activities with repetitive movements increase their risk of upper limb symptoms and RSI [10-12]. However, only a few studies have investigated the prevalence of upper limb symptoms in this high risk group of workers [10,12-14]. We have not located any study in the literature comparing risk factors for clinically diagnosed RSI with the less specific diagnosis of upper limb symptoms.

We studied bank workers of a governmental bank institution in Recife, Northeast Brazil. The aims of the study were: to develop and validate a screening questionnaire for diagnosing RSI; to estimate the prevalence of symptoms of the upper limbs, including those that are potential cases of RSI (which we refer to as having a 'RSI-like condition'), and of clinically confirmed RSI; and to compare some risk factors for RSI with those of 'RSI-like condition' and symptoms of upper limbs.

The comparative description and quantification of upper limb symptoms and RSI among this group of workers at particular risk for the condition adds to the understanding of their epidemiology. It also provides important information for the planning of bank work activities and the design of prevention and control measures for RSI. Of note is that the study tested a simple screening questionnaire for diagnosing cases of RSI, which could be used in similar populations and, subject to further validation, in other populations.

## Methods

We conducted a cross-sectional study between March and August 2000. The reference population consisted of all 579 workers employed by the bank and working at one of the 23 branches located in Recife, all of whom were invited to participate in the study. The type of jobs included managers, cashiers, and administrative clerks. For all these job types, we identified the following ergonomic risk factors: intense use of microcomputers (particularly by the cashiers); continuous work without regular periods of rest; extended working hours; poor posture (elevation of shoulders and elbows, forced rotation of low back, typing while gripping the phone between the head and shoulder); high levels of cognitive demand and constant tension and psychological demands related to expected levels of achievement according to set targets [15].

Managers were responsible for planning and defining targets for local branches; monitoring achievements; decision making and attending special clients. These tasks were carried out using personal computers and telephone, in daily 8 hour shifts. The managers worked seated most of the time.

Cashiers' tasks included dealing with deposits and withdrawals, receiving a wide range of payments and selling branch products for clients. These tasks were performed with the worker standing up for most of their 8 hour shifts, and involved intensive use of personal computers for typing alpha numeric data, and the stamping of many documents (using heavy wooden stamps).

The tasks performed by the clerks varied according to the branches' sectors to which they were allocated. They included liaising with personal and business clients in person and by telephone, a range of administrative activities such as preparing and monitoring contracts for loans and concessions, and checking and typing information onto microcomputers. Their job involved nearly continuous use of personal computers and telephones often simultaneously, although their daily routine varied according to the clients' demands. Compared to the cashiers, they used computers less extensively.

Personnel in other jobs, who were not directly contracted by the bank, were excluded from the study.

### Study procedures

#### Development and validation of the screening questionnaire

A short questionnaire was developed and tested with workers in 2 of the 23 bank branches (n = 41; sub-sample 1). They were self-completed and included four questions on upper limb symptoms aiming to screen individuals with symptoms suggestive of RSI (here referred to as having a 'RSI-like condition'), in addition to addressing personal characteristics of the respondents. A consent form summarizing the study objectives and procedures was appended to the questionnaires and all participants were given a chance to clarify any doubts with the research team. Those who wished to give consent signed the form and returned them with the completed questionnaires.

The specific questions on symptoms of upper limbs covered the clinical definition of RSI (Table 1). A worker with a positive answer to the first question (on report of upper limb symptoms described as sensation of weight, discomfort, weakness or pain) was considered as 'symptomatic'. A worker with a positive answer to all four questions was considered as having a 'RSI-like condition', while symptomatic individuals who did not have a 'RSI-like condition' were considered to have 'symptoms of upper limbs unlikely to be related to RSI'.

**Table 1:** Criteria for diagnosis of RSI included in the screening questionnaire

1. Report of presence of any of the following symptoms in one or both upper limbs: 'sensation of weight', discomfort, weakness or pain in fingers, arms, forearms, elbows or neck
2. Presence of symptom(s) for over one month
3. Presence of symptoms on a daily or nearly daily basis (continuous/recurrent)
4. Relation of symptom(s) with work activities, irrespective of the occurrence of symptoms outside work

The validity of the screening was ascertained by comparing the results of the questionnaire with the clinical diagnosis – used here as the gold standard for the diagnosis of RSI. For that purpose, a full clinical examination was performed in all subjects working in two of the bank branches. Sensitivity, specificity and positive and negative predictive values were derived in the usual way[16].

#### Diagnosis of RSI

The clinical diagnosis was based on the definition of RSI published on the Brazilian Technical Norms for the diagnosis of RSI [17]. Cases of RSI were considered as those meeting the following criteria, based on occupational history and clinical examination:

- Report of regular (daily or nearly daily) symptoms of pain and/or 'paresthesia' in one or both upper limbs for at least one month,

- Relation of work activities with the appearance, intensification or progression of symptoms,

- Absence of other clinical condition that could justify the symptoms described (e.g. diabetes, Hansen's disease, AIDS),

- Presence, in the comparative examination of the upper limbs, of clinical signs related to the symptoms described – including abnormalities on strength or sensitivity, or neurological signs related to specific syndromes (e.g. Tinel's or Phalen's signs in the carpal tunnel syndrome; or Finkelstein's sign in the deQuervain tenosynovitis).

In addition, cases in initial phase were diagnosed based on a typical clinical history with symptoms for over one month, even in the absence of specific clinical signs. These cases correspond to the zero phase of the RSI evolution according the Japanese Association of Occupational Health classification [18].

The diagnosis was established by the Principal Investigator, an occupational physician with large experience in the diagnosis and management of RSI. Medical history taking and clinical examination were conducted 'in situ', i.e. in properly adapted rooms at the bank branches. The clinical examination was made according to standard procedures, including inspection, palpation, passive and active mobilization of the upper limb segments, and focused neurological examination. The results were formally recorded by the research team.

#### Outcomes and exposures

The 'screening questionnaire' was completed by workers in all branches, allowing the ascertainment of the following primary outcomes for the study of risk factors: i) self referred symptoms of upper limbs; ii) self referred symptoms of the upper limbs unlikely to be related to RSI (i.e. symptoms of upper limbs and negative screening for 'RSI-like condition'); iii) 'RSI-like condition'; and iv) clinically confirmed RSI. The latter were identified in a further sub-sample of individuals from all participating branches (n = 64; sub-sample 2) as those with 'RSI-like condition' and a confirmed clinical diagnosis of RSI (as above). We obtained data on potential risk factors, including age, sex, marital status, educational level, occupation and time working at the bank.

### Data handling

All questionnaires were manually checked shortly after completion, and the respondents queried if necessary. Data from the questionnaires and the results of clinical protocols were double entered onto an EPI-info 6.0 database, checked for consistency, and compared with the original records if necessary.

### Data analysis

Data were analysed in EPIINFO 6.0 and STATA version 7. Point prevalence for symptoms of upper limbs, 'RSI-like condition', and confirmed cases of RSI were calculated in standard ways [19].

#### Study of risk factors

The association of exposure variables and symptoms of upper limbs, 'RSI-like condition' and confirmed cases of RSI were tested by the chi-squared test or chi-squared test for trend [19] for categorical variables, or the Wilcoxon rank test for continuous variables (bivariate analysis); and by multiple logistic regression [20]. The multivariate models included age group, sex and all other independent variables associated with the outcome in the bivariate analysis with a significance level of *p *< 0.10. The likelihood ratio test was used to compare models with and without specific independent variables [20], with those which did not contribute to the model (p >= 0.05) not included in the final model. The odds ratio was calculated as a measure of strength of association. We preferred to use this rather than the prevalence rate ratio, as the former is adequate for both the bivariate and the logistic regression analyses. We also specifically tested for the possible interaction between independent variables. A significance level of 0.05 or lower was generally assumed to correspond to a significant result in the bivariate analysis and final multivariate model.

### Sample size

We studied 395 bank workers. This sample size was calculated as adequate to estimate the prevalence of RSI with a precision of 2.5%, assuming a prevalence of 16% in the study population. This figure, which is slightly higher than the 10% prevalence estimated for Brazilian bank workers by Ribeiro [21], was obtained from occupational registries and from the author's experience with bank workers in Recife. The sample size chosen was also adequate to detect associations between risk factors and outcomes with the following odds ratios, with 90% power and a 5% level of significance: 2 and over for symptoms of upper limbs and 'RSI-like condition', and 3 and over for confirmed cases of RSI (calculations made in Stactalc/Epi-info).

### Ethical aspects

The project was approved by the Centro de Pesquisas Aggeu Magalhães Ethics Committee, by the Bank Board of Management, and by the Union of Bank Workers of Pernambuco, the latter representing the workers. All participants signed an informed consent and received feedback on their clinical examinations and were referred to medical care or occupational services as necessary. Participants' anonymity was preserved at all times and their identification was only used for the specific purpose of this research. The ethical principles set by the Declaration of Helsinki were followed.

## Results

The validity of the RSI screening was determined based on the evaluation of all 41 workers from 2 bank branches (sub-sample 1), who completed the questionnaires and were fully assessed clinically (Table 2). Based on a positive clinical diagnosis as the gold-standard for the diagnosis of RSI, we found the screening to have a sensitivity of 90% (9/10) and a specificity of 87% (27/31). The positive and negative predictive values were of 69% and 96% respectively. The observed prevalence of RSI was 24% (10 affected cases out of a total of 41 workers)

**Table 2:** Validity of screening questionnaire for 'RSI-like' cases, showing the clinical diagnosis as the gold standard

Screening Questionnaire	Clinical diagnosis		Total
		
	Case of RSI	Non-case of RSI	
Case of RSI-like (+)	9	4	13
Non-case of RSI-like (-)	1	27	28
Total	10	31	41

Following the validation stage, we invited all bank workers in current activity (n = 579) to participate in the study of prevalence and risk factors. The response rate was 68.9% (n = 399). The analysis refers to 395 subjects (99% of respondents) with complete or near complete information.

The sample consisted of 205 (51.9%) women and 190 (48.1%) men. They were on average 40.4 years old (95% confidence interval (95% CI) = 39.9 – 40.9), and had worked at the bank for a median of 13.1 years. Two hundred and fifty (63.3%) were married or in a stable relationship, 72 (18.2%) were separated, 71 (18%) were single, and 2 (0.5%) widowed. The majority (244 or 82.7%) had a university degree, with 22 of them also having a post-graduate degree. Of those with university degree, 24.3% graduated in business, 16.7% in economy, and 9.9% in law. Most of them worked as either administrative clerks (141 or 36.3%) or cashiers (126 or 32.5%); 76 (19.6%) were managers, and 45 (11.6%) had other jobs.

### Prevalence estimates

We found prevalence rates of 27.1% (n = 102) for symptoms of upper extremities unrelated to RSI, and 29.9% (n = 118) for 'RSI-like condition', giving a total of 56.2% (n = 222) for symptoms of upper extremities. Two 'symptomatic' individuals were excluded due to insufficient information to classify them as unrelated to RSI or 'RSI-like condition'. Considering the cases of 'RSI-like condition' (118/395) and the projection of the validity of the screening to the study population, we estimate a prevalence of RSI in this population of 22% (Table 3).

**Table 3:** Estimated prevalence of RSI in the population based on the validity of the screening instrument

Screening Questionnaire	Clinical diagnosis		Total	Estimated prevalence
			
	+	-		
+	*78*	*40*	**118**	
-	*9*	*268*	*277*	
Total	*87*	*308*	**395**	87/395 = 0.22

### Description of RSI cases

From the clinical exams in the sub-sample 2 (n = 64), we diagnosed 55 workers as true cases of RSI, and 9 as false positive cases. Their main clinical features are shown in Table 4.

**Table 4:** Frequency of features found in clinically diagnosed RSI cases

Features	N	%
Specific diagnostics:		
• De Quervain's tenosynovitis	5	9
• Carpal tunnel syndrome	8	14
• Tenosynovitis of extensors (hands)	1	2
• Tenosynovitis of flexors (hands)	1	2
• Epicondylitis (medial)	8	14
• Epicondylitis (lateral)	7	13
• Biceps tendonitis	3	5
• Supraspinatus tendonitis	9	16
Unspecific signs:		
• Tenderness under palpation	11	20
• Pain under active mobilizing	7	13
• Hypertonus	1	2
• Hyperreflexia	2	4
Diagnosis based on occupational history and symptoms in the absence of clinical signs	15	27

From the 9 false positive cases, 5 reported the symptoms to be no longer present when clinically examined; 2 others reported no symptoms after having changed their jobs within the branch; 1 reported that symptoms disappeared after treatment of a dental chronic infection, and 1 after receiving treatment for tennis elbow and giving up the sport. Latter, we identified two further cases. These cases were originally screened as "negatives" for "RSI-like condition" because they had feared disclosing their condition when answering the 'screening questionnaire'.

### Risk factors for RSI

Table 5 shows the results of the bivariate analyses. Workers with each of the outcomes were compared with workers with no symptoms of upper limbs (n = 173). The variables sex and occupation were significantly associated with all outcomes. Increasing age appeared to confer protection. The variable age-group was significantly associated with symptoms of upper limbs, marginally associated with 'RSI-like condition', but not associated with RSI. None of the other variables tested were significantly associated with any of the outcomes.

**Table 5:** Results of bivariate analyses for RSI and related conditions (4 outcomes), compared to asymptomatic workers (n = 173)

	**Symptoms other than RSI**		**Symptoms**		**'RSI-like'**		**RSI**	
	
**Variables**	**N = 102/275 N (%)**	**OR 95% CI**	**n = 222/395 N (%)**	**OR 95% CI**	**n = 118/291 N (%)**	**OR 95% CI**	**n = 55/228 N (%)**	**OR 95% CI**
Sex:								
Male	40 (39.2)	1*	87 (45.8)	1*	46 (30.9)	1*	18 (14.9)	1*
Female	62 (60.8)	2.28 (1.38 – 3.76)	135 (65.8)	2.28 (1.52 – 3.43)	72 (50.7)	2.30 (1.43 – 3.72)	37 (3.0)	3.02 (1.60 – 5.74)
Job:								
Manager	19 (18.1)	1	32 (42.1)	1^†^	13 (22.8)	1^†^	6 (12.0)	1^†^
Clerk	31 (30.7)	1.20 (0.60 – 2.39)	81 (57.4)	1.86 (1.04 – 3.29)	50 (45.4)	2.85 (1.40 – 6.28)	28 (31.8)	3.69 (1.35 – 10.09)
Cashier	41 (40.6)	2.16 (1.09 – 4.28)	82 (65.1)	2.56 (1.40 – 4.67)	39 (47.0)	2.43 (1.20 – 5.65)	18 (29.0)	2.60 (0.92 – 7.38)
Other	10 (9.1)	1.10 (0.44 – 2.78)	24 (53.3)	1.57 (0.74 – 3.32)	14 (40.0)	1.69 (0.88 – 5.76)	2 (8.7)	0.70 (0.13 – 3.76)
Education:								
Up to secondary school	33 (32.4)	1	82 (56.2)	1	48 (42.8)	1	22 (25.5)	1
University degree	69 (67.8)	1.23 (0.73 – 2.06)	140 (56.2)	1.00 (0.66 – 1.51)	70 (39.1)	1.52 (0.84 – 2.78)	33 (23.2)	1.24 (0.59 – 2.62)
Marital status:								
Single	24 (23.5)	1	41 (57.8)	1	16 (34.8)	1	6 (16.7)	1
Married	58 (56.9)	0.65 (0.35 – 1.22)	139 (55.6)	0.92 (0.54 – 1.56)	80 (41.9)	1.35 (0.69 – 2.64)	42 (27.4)	1.89 (0.73 – 4.87)
Separated/Divorced	20 (19.6)	0.83 (0.38 – 1.82)	42 (56.8)	1.02 (0.53 – 1.99)	22 (40.7)	1.38 (0.61 – 3.12)	7 (18.0)	1.17 (0.35 – 3.88)

	**Mean Diff (SE)**	**OR 95%CI**	**Mean Diff (SE)**	**OR 95%CI**	**Mean diff (SE)**	**OR 95%CI**	**Mean diff (SE)**	**OR 95%CI**

Age (OR by 5 year age-groups)	1.59 (0.67)	0.75 (0.58 – 0.96)^†^	1.46 (0.53)	0.77 (0.63 – 0.95)^∞^	1.34 (0.62)	0.80 (0.62 – 1.02)	0.96 (0.82)	0.82 (0.60 – 1.12)
Time at bank in years (OR by year worked in bank)	1.29 (0.65)	0.95 (0.91 – 1.00)^∞^	1.04 (0.51)	0.96 (0.92 – 1.0)	0.89 (0.61)	0.97 (0.92 – 1.01)	0.30 (0.81)	0.99 (0.93 – 1.05)

* *p *< 0.001, ^† ^*p *< 0.02, ^∞ ^*p *<= 0.05

'Symptoms other than RSI" refers to individuals considered to have 'symptoms of upper limbs unlikely to be related to RSI' (n = 102).

The variable 'symptoms' refers to all subjects with symptoms of upper extremities (n = 222).

To be considered as having 'RSI-like condition', positive responses to all 4 screening questions were required (n = 118).

RSI includes confirmed cases of RSI following full clinical assessment (n = 55).

For the variable symptoms, the total sample size of 375 is used as the denominator.

For the other symptom-related variables, the denominator includes 173 cases with no symptoms of upper limbs added to the respective numerator.

In the logistic regression analysis, sex and occupation remained significantly associated with all outcomes (Table 6). Female sex was directly associated with all outcomes, with odds ratios ranging from 2.2 to 3.1 and a stronger association for confirmed cases of RSI. The highest occupational risk for symptoms of upper limbs was for cashiers, while those for 'RSI-like condition' and RSI were for clerks. While the strength of association for cashiers increased slightly though consistently from the less to the more specific outcomes, the risk of clerks increased more dramatically, from 1.4 for symptoms of upper limbs other than due to RSI, up to 3.7 in confirmed cases of RSI. Age-group, as a continuous variable, was a significant predictor of symptoms other than due to RSI (p = 0.05), marginally significant (p = 0.07) for the outcome 'symptoms of upper limbs' – with a trend for protection with increasing age, but became non-significant for the two other outcomes (Table 6).

**Table 6:** Results of logistic regression analyses for RSI and related conditions (4 outcomes); final model

**VARIABLES**	**Symptoms other than RSI OR 95% CI**	**Symptoms OR 95% CI**	**'RSI-like' OR 95% CI**	**RSI OR 95% CI**
Sex:				
Male	1*	1*	1***	1***
Female	2.31 (1.37 – 3.89)	2.24 (1.47 – 3.53)	2.27 (1.36 – 3.79)	3.14 (1.58 – 6.28)
Job:				
Manager	1**	1**	1*	1***
Clerk	1.45 (0.70 – 3.0)	2.02 (1.11 – 3.66)	2.85 (1.40 – 6.28)	3.69 (1.35 – 10.09)
Cashier	2.12 (1.04 – 4.31)	2.40 (1.31 – 4.41)	2.43 (1.20 – 5.65)	2.61 (0.92 – 7.38)
Other	0.97 (0.37 – 2.53)	1.48 (0.69 – 3.20)	1.69 (0.88 – 5.76)	0.65 (0.12 – 3.61)
Age in 5 year periods	0.76 (0.59 – 1.0)	0.82 (0.66 – 1.01)	0.86 (0.66 – 1.12)	0.89 (0.62 – 1.26)

* *p *< 0.001, ** *p *< 0.02, *** *p *< 0.01

'Symptoms other than RSI" refers to individuals considered to have 'symptoms of upper limbs unlikely to be related to RSI' (n = 102).

The variable 'symptoms' refers to all subjects with symptoms of upper extremities (n = 222).

To be considered as having 'RSI-like condition', positive responses to all 4 screening questions were required (n = 118).

RSI includes confirmed cases of RSI following full clinical assessment (n = 55).

## Discussion

This is the first study investigating the prevalence of RSI in a population of bank workers in Northeast Brazil. The diagnosis of 'RSI-like condition' was made by a simple screening questionnaire based on the definition of RSI, which is easy to apply and has high sensitivity and specificity. Pending further validation in other settings, this could become a widely used instrument in the study of RSI. Diagnostic confirmation and further categorization of cases should be done using clinically available guidelines [22]. We believe the clinical diagnosis of RSI in the study to be very reliable, as it was made by an experienced clinician based on standard procedures. This included a detailed history and physical examination, with exclusion of other conditions that could justify the symptoms in the upper limbs. Nevertheless, the diagnosis is still subject to misclassification, particularly in early or very mild cases of RSI that lack clinical signs. However, in the absence of a pathognomonic diagnostic test, we believe this to be the best means of diagnosing true cases of RSI and of other upper limb conditions. Other screening tests have been proposed to musculoskeletal symptoms related to occupation [14,22-24]. We used a shorter instrument with only 4 questions focusing on upper limb symptoms, which had good sensitivity (90%) and specificity (87%). The false negatives occurred mainly as a result of fear on the part of subjects to disclose their condition. A similar situation was reported by the British RSI association, who claimed "the majority of people experiencing pain, discomfort and loss of function due to musculoskeletal problems in the workplace make no reference to their condition for fear of losing their job"[25]. False positives were mainly due to other temporary conditions of the upper limbs. Such cases can easily be distinguished from true cases of RSI by full clinical assessment, which can be repeated if necessary after a short interval.

The frequency of specific RSI-related diagnoses was crudely similar across anatomic regions (hands and wrists, elbows and shoulders/neck). Carpal tunnel syndrome was unsurprisingly the most frequent specific diagnosis related to RSI, as previously reported in literature [26-29]. This arises due to the specific ergonomic conditions at work sites, e.g. hyperflexion of wrists while typing and other poor postures at work, which may be more important among cashiers than in managers. However, we found a similarly high frequency of epicondylitis. The full understanding of these findings would require further research, including the use of ergonomic techniques.

The literature has been inconsistent on the outcomes studied; some report on work related symptoms of upper limbs [30,31], others refer to more specific diagnoses such as work related carpal tunnel syndrome [28,29,32-36], or to an occupational syndromic condition such as RSI [3,37-41]. It is not clear whether these outcomes are truly comparable, and it is possible that they have different determinants. In this study, we compared some risk factors for a) upper limb symptoms; b) 'RSI-like condition', defined by suggestive symptoms, and c) clinically diagnosed RSI. Although female sex and work as a cashier or clerk were associated with the 3 outcomes, the strength of these associations increased as more specific diagnoses of 'RSI-like condition' and RSI were used. This suggests that while female sex is a risk factor for symptoms of upper limbs, as reported consistently in the literature [14,42-52], much of this risk is accounted for in true cases of RSI, with other cases of symptoms of upper limbs having a weaker association with female sex.

Workers who develop symptoms of RSI and especially those with clinically confirmed diagnosis tend to change their roles within the organisation. Very often this means changing from cashiers to clerks, with the objective of becoming less exposed to repeated movements. Reverse causality may therefore partially explain the higher risk of clerks for RSI than for other upper limb conditions.

Age was significantly associated with symptoms of upper limbs, but not with 'RSI-like condition' or RSI. This suggests increasing age to be protective of conditions other than RSI that present with upper limb symptoms, but caution is warranted when interpreting these findings. Young age may be linked to other problems affecting upper limbs in relatively healthy individuals, such as trauma and sports injuries. Change to lower risk jobs as workers age, 'healthy worker' effects and uncontrolled confounding may also explain these findings. It is also possible that young workers belong to a cohort of individuals that share common risk factors to symptoms of upper limbs not explored here. If this represents a cohort effect, then this would be consistent with an increasing incidence of upper limb conditions. While this is speculative, taken the cross-sectional nature of this study, this confirms a clinical impression of there being an increasing problem in the study area and indeed in other settings. The weakening of the association with age, as we move from symptoms of upper limbs to RSI, could also be interpreted as being due to less specific symptoms representing early stages of RSI in younger individuals, which develop in some of them into full-blown RSI as they age. This would be supported by the age distribution of the sample, with non-RSI related symptoms affecting the younger individuals (mean age of 39.6 years) and confirmed RSI affecting those on average 41.0 years old, with RSI-like condition having intermediate mean ages of 39.9 years. Nevertheless, in contrast to other studies [2,10,12,14,25,29,35,42,53-58], true cases of RSI were not found to be significantly associated with age. The use of a less specific diagnosis in other studies and variation in populations may explain these differences.

We estimated the prevalence of RSI as 22% and that of symptoms of upper limbs as 56%, for this particularly high risk group. Population based prevalence figures for RSI have been typically lower. In Canada, 10% of the population over 20 years old reported RSI serious enough to limit usual activities at some point in the previous 12 months [45]. In the Netherlands, the population prevalence in individuals over 25 years old has been estimated as around 2% for RSI, 11% for epicondylitis, and 16% for tendonitis or capsulitis [44]. Higher prevalence rates have been reported among specific occupational groups, e.g. with prevalence over 60% of pain [57] or musculoskeletal complains of the upper limbs [31] reported in dentists. In bank workers, some researchers reported prevalence of upper limb symptoms varying according to the affected anatomic region e.g. from 6.6% for symptoms in arms to 31.4% in neck [12], and from 16% in elbow to 50% in shoulder [14]. In Southeast Brazil, the prevalence of RSI among bank workers has been reported as 10% with another 10% presenting upper limb symptoms, but not a diagnosis of RSI [21]. We found relatively high prevalence rates of upper limb symptoms and RSI, indicating perhaps a particularly high risk population in our study. The prevalence found was also higher than that predicted based on the author's previous experience as an occupational physician. A possible reason is that many individuals affected by RSI continue to carry out their work activities in spite of the symptoms and do not see the occupational doctor. However, it should be noted that the prevalence of RSI is particularly high among workers at this bank and a more comprehensive study of risk factors in this population could give further insights on why this is the case. The prevalence of RSI was based on the assumed validity of the screening questionnaire for cases of 'RSI-like condition'. As these estimates are subject to random error, this may have lead to some imprecision in the prevalence estimates.

We were not able to include a considerable proportion of the workers in the study. Workers with RSI who were on sick leave, and who probably represent the most severe cases, were excluded from the study due to logistic and ethical reasons. If a relatively large number of RSI cases are likely to be on sickness related absences, this may have led to an underestimation of the prevalence of RSI, and possibly also an underestimation of the association of independent variables with the outcomes. Similarly, if workers with RSI were less likely to participate so as to avoid disclosing their condition (with fear that this might affect their jobs), this would have similar effects.

Only a small selection of variables was used in the analysis. This excluded, for example, variables related to the work organization and environment, which may confound the associations investigated in the study. A comprehensive report on determinants of RSI and related conditions was not, however, the aim of this paper.

The study was conducted in a specific bank in Northeast Brazil. The results are representative of this specific population, but probably also of bank workers in general, particularly those who are subject to similar working conditions.

## Conclusion

In conclusion, our study showed a high prevalence of RSI and related conditions in this population of bank workers, raising serious concerns about the magnitude of potentially disabling conditions in this occupational group, and calling for urgent measures to improve work environments and how they are organised. It also confirms gender and certain specific occupational roles in the risk of RSI and related conditions, with stronger associations found among confirmed cases of RSI. The results also suggest age to be more directly linked to symptoms of upper limbs that are not related to RSI. The variable magnitude of the associations suggests that risk factors differ slightly according to the definition/outcome used, and raises a question on the comparability of previous studies using different diagnostic criteria. Further prospective studies with the inclusion of a larger number of potential risk factors would help clarify the role of these and other variables in the aetiology of RSI. We propose the use of a simple screening questionnaire to identify potential cases of RSI, namely cases of 'RSI-like condition'. Pending further validation, the use of such a questionnaire, complemented by full clinical evaluation is a sensible way to identify cases of RSI for population based epidemiological studies in a consistent way.

## Competing interests

The author(s) declare that they have no competing interests.

## Authors' contributions

EML participated in the conception and study design, carried out the clinical examinations, participated in the analysis and interpretation of data, and has been involved in the drafting the article. LCN participated in the conception and design of the study, in the analysis and interpretation of data, and has been involved in the drafting the article. LASG participated in the conception and study design, and critically revised the article. MTO participated in the interpretation of data and critically revised the article. DCFR was involved in the acquisition of data and critically revised the article. DCAW has been involved in the acquisition of data and revised critically the article. All authors approved the manuscript.

## Pre-publication history

The pre-publication history for this paper can be accessed here:


